# Health profession education hackathons: a scoping review of current trends and best practices

**DOI:** 10.1186/s12909-024-05519-7

**Published:** 2024-05-21

**Authors:** Azadeh Rooholamini, Mahla Salajegheh

**Affiliations:** Department of Medical Education, Medical Education Development Center, Kerman University of Medical Sciences, Kerman, Iran

**Keywords:** Hacking, Education, Medical education, Health profession education, Innovation

## Abstract

**Background:**

While the concept of hacking in education has gained traction in recent years, there is still much uncertainty surrounding this approach. As such, this scoping review seeks to provide a detailed overview of the existing literature on hacking in health profession education and to explore what we know (and do not know) about this emerging trend.

**Methods:**

This was a scoping review study using specific keywords conducted on 8 databases (PubMed, Embase, Scopus, Web of Science, ERIC, PsycINFO, Education Source, CINAHL) with no time limitation. To find additional relevant studies, we conducted a forward and backward searching strategy by checking the reference lists and citations of the included articles. Studies reporting the concept and application of hacking in education and those articles published in English were included. Titles, abstracts, and full texts were screened and the data were extracted by 2 authors.

**Results:**

Twenty-two articles were included. The findings are organized into two main categories, including (a) a Description of the interventions and expected outcomes and (b) Aspects of hacking in health profession education.

**Conclusion:**

Hacking in health profession education refers to a positive application that has not been explored before as discovering creative and innovative solutions to enhance teaching and learning. This includes implementing new instructional methods, fostering collaboration, and critical thinking to utilize unconventional approaches.

**Supplementary Information:**

The online version contains supplementary material available at 10.1186/s12909-024-05519-7.

## Introduction

Health professions education is a vital component of healthcare systems to provide students with the knowledge, skills, and attitudes necessary to provide high-quality care to patients [1]. However, with the advent of innovative technologies and changing global dynamics, there is a growing need to incorporate new educational methods to prepare medical science students for the future [2].

Although traditional methods can be effective for certain learning objectives and in specific contexts and may create a stable and predictable learning environment, beneficial for introducing foundational concepts, memorization, and repetition, however, they may not fully address the diverse needs and preferences of today’s learners [3]. Some of their limitations may be limited engagement, passive learning, lack of personalization, and limited creativity and critical thinking [4].

As Du et al. (2022) revealed the traditional teaching model fails to capture the complex needs of today’s students who require practical and collaborative learning experiences. Students nowadays crave interactive learning methods that enable them to apply theoretical knowledge in real-world situations [5].

To achieve innovation in health professions education, engaging students and helping them learn, educators should use diverse and new educational methods [6]. Leary et al. (2022) described how schools of nursing can integrate innovation into their mission and expressed that education officials must think strategically about the knowledge and skills the next generation of students will need to learn, to build an infrastructure that supports innovation in education, research, and practice, and provide meaningful collaboration with other disciplines to solve challenging problems. Such efforts should be structured and built on a deliberate plan and include curricular innovations, and experiential learning in the classroom, as well as in practice and research [7].

The incorporation of technology in education is another aspect that cannot be ignored. Technology has revolutionized the way we communicate and learn, providing opportunities for students to access information and resources beyond the traditional education setting. According to the advancement of technology in education, hacking in education is an important concept in this field [8].

Hack has become an increasingly popular term in recent years, with its roots in the world of computer programming and technology [9]. However, the term “hack” is not limited solely to the realm of computers and technology. It can also refer to a creative approach to problem-solving, a willingness to challenge established norms, and a desire to find new and innovative ways to accomplish tasks [10]. At its core, hacking involves exploring and manipulating technology systems to gain a deeper understanding of how they work. This process of experimentation and discovery can be applied to many different fields, including education [11].

In education, the concept of “hack” has become popular as educators seek innovative ways to engage students and improve learning outcomes. As Wizel (2019) described “hack in education” involves applying hacker mentality and techniques, such as using technology creatively and challenging traditional structures, to promote innovation within the educational system [12]. These hacking techniques encompass various strategies like gamification, hackathons, creating new tools and resources for education, use of multimedia presentations, online forums, and educational apps for project-based learning [9]. Butt et al. (2020) demonstrated the effectiveness of hack in education in promoting cross-disciplinary learning in medical education [13]. However, concerns exist about the negative connotations and ethical implications of hacking in education, with some educators hesitant to embrace these techniques in their classrooms [7, 14].

However, while the concept of hack in education has gained traction in recent years, there is still a great deal of uncertainty surrounding its implementation and efficacy. As such, this scoping review seeks to provide a comprehensive overview of the existing literature on hacking in health profession education (HPE), to explore what we know (and do not know) about this emerging trend. To answer this research question, this study provided a comprehensive review of the literature related to hacking in HPE. Specifically, it explored the various ways in which educators are using hack techniques to improve learning outcomes, increase student engagement, and promote creativity in the classroom.

## Methods and materials

This scoping review was performed based on the Arksey and O’Malley Framework [15] and Preferred Reporting Items for Systematic Reviews and Meta-Analyses (PRISMA) statement to answer some questions about the hacking approach in health professions education [16].

### Search strategies

The research question was “What are the aspects of hacking in education?“. We used the PCC framework which is commonly used in scoping reviews to develop the research question [17]. In such a way the Population assumed as learners, the Concept supposed as aspects of hacking in education, and the Context is considered to be the health profession education.

A systematic literature search was conducted on June 2023, using the following terms and their combinations: hack OR hacking OR hackathon AND education, professional OR “medical education” OR “medical training” OR “nursing education” OR “dental education” OR “pharmacy education” OR “health professions education” OR “health professional education” OR “higher education” OR “healthcare education” OR “health care education” OR “students, health occupations” OR “medical student” OR “nursing student” OR “dental student” OR “pharmacy student” OR “schools, health occupations” OR “medical school” OR “nursing school” OR “dental school” OR “pharmacy school”) in 8 databases (PubMed, Embase, Scopus, Web of Science, ERIC, PsycINFO, Education Source, CINAHL) with no time limitation. (A copy of the search strategy is included in Appendix 1). To find additional relevant studies, we conducted a forward and backward searching strategy by checking the reference lists and citations of the included articles.

### Inclusion and exclusion criteria

Original research reporting the different aspects of hacking in health professions education and published in English was included. We excluded commentaries, editorials, opinion pieces, perspectives, reviews, calls for change, needs assessments, and other studies in which no real interventions had been employed.

### Study identification

After removing the duplicates, each study potentially meeting the inclusion criteria was independently screened by 2 authors (A.R. and M.S.). Then, the full texts of relevant papers were assessed independently by the 2 authors for relevance and inclusion. Disagreements at either step were resolved when needed until a consensus was reached.

### Quality assessment of the studies

We used the BEME checklist [18], consisting of 11 indicators, to assess the quality of studies. Each indicator was rated as “met,” “unmet,” or “unclear.” To be deemed of high quality, articles should meet at least 7 indicators. The quality of the full text of potentially relevant studies was assessed by 2 authors (A.R. and M.S.). Disagreements were resolved through discussion. No study was removed based on the results of the quality assessment.

### Data extraction and synthesis

To extract the data from the studies, a data extraction form was designed based on the results of the entered studies. A narrative synthesis was applied as a method for comparing, contrasting, synthesizing, and interpreting the results of the selected papers. All outcomes relevant to the review question were reported. The two authors reviewed and coded each included study using the data extraction form independently.

## Results

**Fig. 1:**
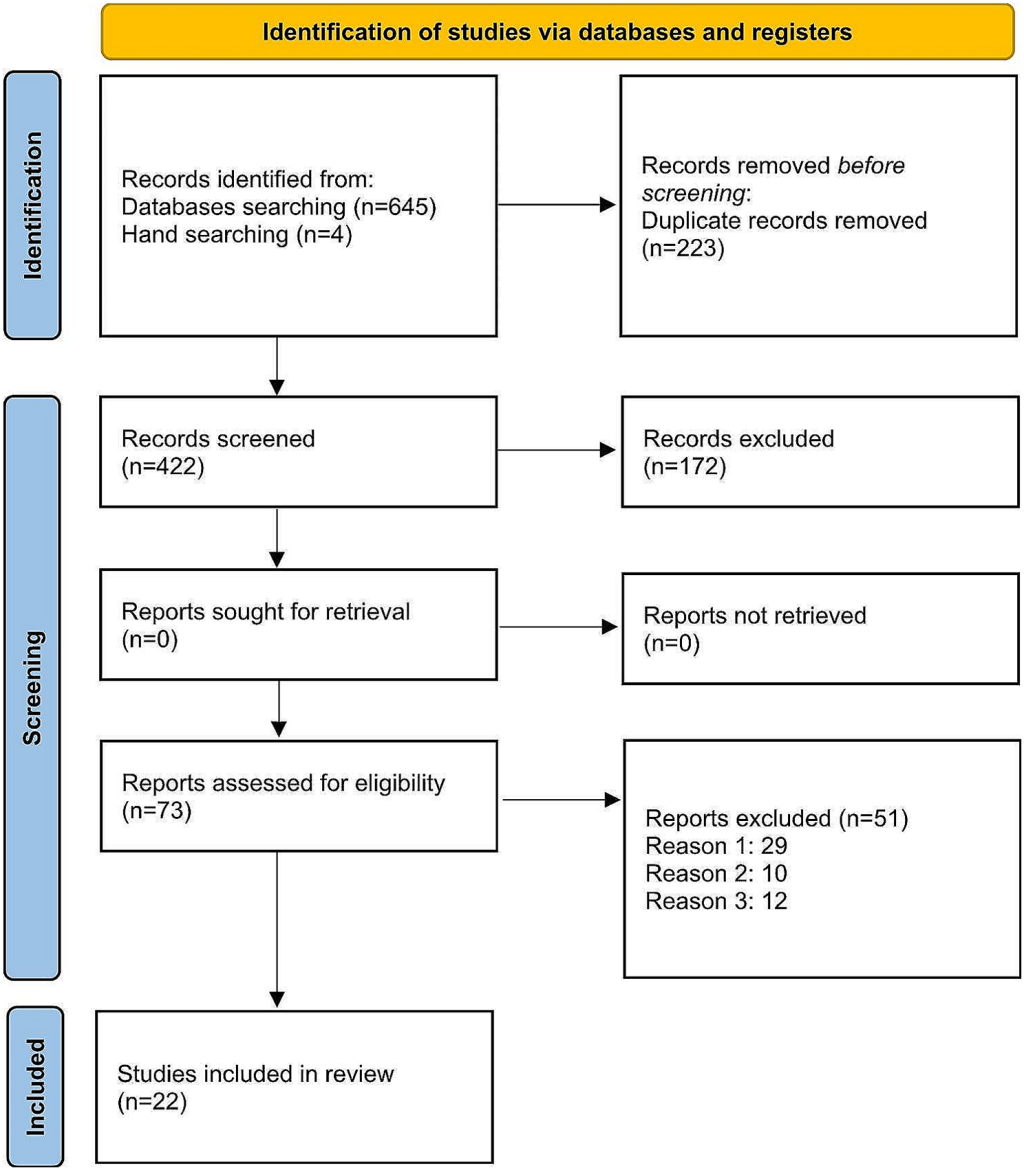
PRISMA flow diagram for included studies

A total of 645 titles were found, with a further four titles identified through the hand-searching of reference lists of all reviewed articles. After removing the duplicate references, 422 references remained. After title screening, 250 studies were considered for abstract screening, and 172 studies were excluded. After the abstract screening, 73 studies were considered for full-text screening, and 177 studies were excluded due to reasons such as:1. being irrelevant, 2. loss of data, and 3. language limitation. 22 studies were included in the final analysis. The 2020 PRISMA diagram for the included studies is shown in Fig. 1. The quality was evaluated as “high” in 12 studies, “moderate” in 7 studies, and “low” in 3 studies.

The review findings are organized into two main categories: (a) Description of the interventions and expected outcomes and (b) Aspects of hacking in health profession education.

### Description of the interventions and expected outcomes

The description of the studies included the geographical context of the interventions, type, and number of participants, focus of the intervention, evaluation methodology, and outcomes. Table 1 displays a summary of these features.

#### Geographical context

Of the 22 papers reviewed, 11 studies (45.4%) took place in the United States of America [7, 19–28], two studies in Pakistan [13, 29], one study performed in international locations [30], and the remainder being in the United Kingdom [31], Germany [32], Finland [33], Australia [34], Austria [35], Thailand [36], Africa [37], and Canada [38].

#### Type and number of participants

Hacking in HPE interventions covered a wide range and multiple audiences. The majority of interventions targeted students (17 studies, 77.2%) [7, 13, 20, 21, 23–27, 29–33, 36–38]. Their field of education was reported differently including medicine, nursing, engineering, design, business, kinesiology, and computer sciences. Also, they were undergraduates, postgraduates, residents, and post-docs. Ten interventions (45.4%) were designed for physicians [13, 19, 21, 24–26, 28, 29, 33, 35]. Their field of practice was reported diverse including psychology, radiology, surgery, and in some cases not specified. Eight (36.3%) studies focused on staff which included healthcare staff, employees of the university, nurses, care experts, and public health specialists [13, 22, 26, 28–30, 32, 35]. Interestingly, nine of the hacking in HPE interventions (40.9%) welcomed specialists from other fields outside of health sciences and medicine [13, 19, 22, 25, 26, 28, 29, 33, 35]. Their field of practice was very diverse including engineers, theologians, artists, entrepreneurs, designers, informaticists, IT professionals, business professionals, industry members, data scientists, and user interface designers. The next group of participants was faculty with 5 studies (22.7%) [7, 23, 32, 34, 36]. An intervention (4.5%) targeted the researchers [27]. The number of participants in the interventions ranged from 12 to 396. Three studies did not specify the number of their participants.

#### The focus of the intervention

The half of interventions aimed to improve HPE (12 studies, 54.5%) [7, 13, 21, 23, 24, 26, 28, 30–32, 34, 38], with a secondary emphasis on enhancing clinical or health care [19, 22, 25, 29, 33, 35–37]. Two studies highlighted the improvement in entrepreneurship skills of health professions [19, 20]. One study aimed to improve the research skills of health professionals [27].

#### Evaluation methodology

Methods to evaluate hacking in HPE interventions included end-of-program questionnaires, pre-and post-test measures to assess attitudinal or cognitive change, self-assessment of post-training performance, project-based assessment through expert judgment and feedback, interviews with participants, and direct observations of behavior.

#### Outcomes

Hacking in HPE interventions has resulted in positive outcomes for participants. Five studies found high levels of satisfaction for participants with the intervention [21, 31–33, 37]. Some studies evaluated learning, which included changes in attitudes, knowledge, and skills. In most studies, participants demonstrated a gain in knowledge regarding awareness of education’s strengths and problems, in the desire to improve education by enhancement of awareness for technological possibilities [7, 13, 19, 21, 23, 30, 32–35, 38]. Some studies found improving participant familiarity with healthcare innovation [19, 22, 24–26, 33, 36, 37]. Some participants reported a positive change in attitudes towards HPE as a result of their involvement in hacking interventions. They cited a greater awareness of personal strengths and limitations, increased motivation, more confidence, and a notable appreciation of the benefits of professional development [20, 21, 29, 34]. Some studies also demonstrated behavioral change. In one study, changes were noted in developing a successful proof-of-concept of a radiology training module with elements of gamification, enhancement engagement, and learning outcomes in radiology training [28]. In a study, participants reported building relationships when working with other members which may be students, faculty, or healthcare professionals [7]. Five studies found a high impact on participant perceptions and attitudes toward interdisciplinary collaboration [22, 26, 27, 36, 38].

### Aspects of hacking in health profession education

The special insights of hacking in HPE included the adaptations considered in the interventions, the challenges of interventions, the suggestions for future interventions, and Lessons learned.

#### Adaptations

The adaptations are considered to improve the efficacy of hacking in HPE interventions. We found that 21 interventions were described as hackathons. Out of this number, some were only hackathons, and some others had benefited from hackathons besides other implications of hacking in education. Therefore, most of the details in this part of the findings are presented with a focus on hackathons. The hackathon concept has been limited to the industry and has not been existing much in education [39, 40]. In the context of healthcare, hackathons are events exposing healthcare professionals to innovative methodologies while working with interdisciplinary teams to co-create solutions to the problems they see in their practice [19, 22, 24, 25, 30, 41, 42].

Some hackathons used various technologies for internal and external interactions during the hackathon including Zoom, Gmail, WhatsApp, Google Meet, etc [37]. . . Almost all hackathons were planned and performed in the following steps including team formation, team working around the challenges, finding innovative solutions collaboratively, presenting the solutions and being evaluating based on some criteria including whether they work, are good ideas with a suitable problem/solution fit, how a well-designed experience and execution, etc. For example, in the hackathon conducted by Pathanasethpong et al. (2017), the judging criteria included innovativeness, feasibility, and value of the projects [36]. Also, they managed the cultural differences between the participants through strong support of leadership, commitment, flexibility, respect for culture, and willingness to understand each other’s needs [36].

#### Challenges

Despite valuable adaptations, several challenges were reported. The hackathons faced some challenges such as limited internet connectivity, time limitations, limited study sample, power supply, associated costs, lack of diversity among participants, start-up culture, and lack of organizational support [13, 19, 25, 28, 30, 34, 37]. Some interventions reported the duration of the hackathon was deemed too short to develop comprehensive solutions [37]. One study identified that encouraging experienced physicians and other healthcare experts to participate in healthcare hackathons is an important challenge [26].

#### Suggestions for the future

Future hackathons should provide internet support for participants and judges, invite investors and philanthropists to provide seed funding for winning teams, and enable equal engagement of all participants to foster interdisciplinary collaboration [37]. Subsequent hackathons have to evaluate the effect of implementation or durability of the new knowledge in practice [19, 28]. Wang et al. (2018) performed a hackathon to bring together interdisciplinary teams of students and professionals to collaborate, brainstorm, and build solutions to unmet clinical needs. They suggested that future healthcare hackathon organizers a balanced distribution of participants and mentors, publicize the event to diverse clinical specialties, provide monetary prizes and investor networking opportunities for post-hackathon development, and establish a formal vetting process for submitted needs that incorporates faculty review and well-defined evaluation criteria [22]. Most interventions had an overreliance on self-assessments to assess their effectiveness. To move forward, we should consider the use of novel assessment methods [30].

#### Lessons learned

Based on the findings of hackathons, they have developed efficient solutions to different problems related to public health and medical education. Some of these solutions included developing novel computer algorithms, designing and building model imaging devices, designing more approachable online patient user websites, developing initial prototypes, developing or optimizing data analysis tools, and creating a mobile app to optimize hospital logistics [25–27, 36]. Staziaki et al. (2022) performed an intervention to develop a radiology curriculum. Their strategies were creating new tools and resources, gamification, and conducting a hackathon with colleagues from five different countries. They revealed a radiology training module that utilized gamification elements, including experience points and a leaderboard, for annotation of chest radiographs of patients with tuberculosis [28].

Most hackathons provide an opportunity for medical health professionals to inter-professional and inter-university collaboration and use technology to produce innovative solutions to public health and medical education [7, 23, 26, 30, 37, 38]. For example, one study discussed that hackathons allowed industry experts and mentors to connect with students [37]. In the study by Mosene et al. (2023), results offer an insight into the possibilities of hackathons as a teaching/learning event for educational development and thus can be used for large-scale-assessments and qualitative interviews for motivational aspects to participate in hackathons, development of social skills and impact on job orientation [32].

The participants’ willingness to continue working on the projects after the hackathons was also reported in some papers [13, 29, 33]. One study highlights the potential of hackathons to address unmet workforce needs and the preference of female surgeons for small-group discussions and workshops [24]. Craddock et al. (2016) discussed that their intervention provided a unique opportunity for junior researchers and those from developing economies who have limited opportunities to interact with peers and senior scientists outside their home institution [27].

Dameff et al. (2019) developed and evaluated a novel high-fidelity simulation-based cybersecurity training program for healthcare providers. They found significant improvements in the knowledge and confidence of participants related to clinical cybersecurity after completing the simulation exercise. They also reported high levels of satisfaction with the training program [21].

**Table 1:** Summary of hacking in Health Professions Education interventions reviewed

Num	First author	Publication	Journal	Geographical location	Timeframe	Type of study	Number of participants	Type of participants	Focus of the intervention	Evaluation methodology	Outcomes	Implications of hack in education	Quality of study
1	Marion Leary [7]	December 2021	Journal of Professional Nursing	United States of America	-	Qualitative	-	Faculty and students	Providing a case study of how to infuse innovation into a school of nursing	Qualitatively/ questionnaire	Faculty gained knowledge and built relationships when working with students and learned about innovation	Creating new tools and resources	Moderate
2	Waqaas Akmal Butt [13]	October 2020	BMJ Innovations	Pakistan	12 months	Mixed methods	116	Students, physicians, healthcare staff, IT engineers	Developing a hackathon as an educational tool for technical and entrepreneurial skills for undergraduate and postgraduate medical education and evaluating its effectiveness	Quantitatively/ questionnaire. Qualitatively/ focused group	Hackathons are considered an alternative and cross-disciplinary teaching and learning tool.	Hackathon	High
3	Hanna Kienzler [31]	February 2017	Teaching in Higher Education	United Kingdom	10 weeks	Mixed methods	36	Students	Developing a hackathon for inquiry-based learning and evaluating its effectiveness	Quantitatively/ questionnaire. Qualitatively/ feedback	Students had a positive learning experience	Hackathon	High
4	Katharina Mosene [32]	March 2023	GMS Journal for Medical Education	Germany	3 months	Quantitative	60	Faculty, students, and employees of the university	Developing a hackathon to tackle current problems in education and evaluating its effectiveness	Quantitively/ questionnaire	The results of the use of hackathons as educational development tools were positive. Results offer possibilities of hackathons as a teaching/learning event for educational development	Hackathon	High
5	Emmanuel Awuni Kolog [33]	June 2016	International Journal of Modern Education and Computer Science	Finland	3 months	Mixed methods	12	Students, a medical doctor, a theologian, and an artist	Developing a hackathon to design an application aimed at people that are preparing for their death and evaluating their effectiveness	Qualitatively/ interview. Quantitively/ questionnaire	Achieving the learning goals. High satisfaction of students, their more motivation	Hackathon	High
6	Jason K. Wang [30]	October 2018	Journal of Medical Systems	International locations	June 2015-May 2018	Quantitative	245	Students and health professionals	Developing a hackathon to make medical innovation education and training more accessible and easily adaptable for academic medical centers and evaluating its effectiveness	Quantitively/ questionnaire	Gaining significant knowledge	Hackathon	High
7	Gabrielle Brand [34]	November 2020	Medical Teacher	Australia	2 months	Mixed methods	163	Faculty	Developing a hackathon to teach sustainable healthcare education and evaluating its effectiveness	Quantitatively/ questionnaire. Qualitatively/ interview	Gaining strong content knowledge and more confidence	Hackathon	High
8	Carl Preiksaitis [19]	February 2023	JMIR Medical Education	United States of America	3 days	Qualitative	24	Physicians, engineers, entrepreneurs, designers	Developing a hackathon to improve emergency physician familiarity with the principles of healthcare innovation and entrepreneurship and exploring the learning experience of participants	Qualitatively/ interview	Improving participant familiarity with healthcare innovation and teaching entrepreneurship	Hackathon	Moderate
9	Olga Kagan [20]	February 2023	Nursing Outlook	United States of America	2 months	Quantitative	396	Nurses, students	Developing a hackathon to improve confidence levels in starting a new venture, startup, or project in nursing and entrepreneurship and evaluating its effectiveness	Quantitively/ questionnaire	Increasing confidence levels as innovators	Hackathon	High
10	Daniela E. Ströckl [35]	May 2022	IndHealth	Austria	2 days	-	15	Psychologists, engineers, care experts	Developing a hackathon to support and integrate young care students into the care digitization process	-	Enhancement of awareness for technological possibilities and empower future care workers	Hackathon	Low
11	Pedro Vinicius Staziaki [28]	February 2022	Journal of Digital Imaging	United States of America	5 days	Quantitative	14	Radiologists, informaticists, healthcare professionals	Creating a radiology training module that incorporated gamification elements to enhance engagement and learning outcomes	Quantitively/ questionnaire	A successful proof-of-concept of a radiology training module with elements of gamification. Enhancement engagement and learning outcomes in radiology training	Creating new tools and resources/ Gamification/ Hackathon	Moderate
12	Christian J. Dameff [21]	February 2019	Journal of Emergency Medicine	United States of America	-	Qualitative	13	Students, physicians	Developing and evaluating a novel high-fidelity simulation-based cybersecurity training program for healthcare providers	Qualitatively/ Feedback, reflection	Significant improvements in knowledge and confidence related to clinical cybersecurity. High levels of satisfaction with the program	Simulation-based training	Moderate
13	Jason K. Wang [22]	December 2018	BMC Medical Education	United States of America	6 months	Quantitative	257	Engineers, designers, entrepreneurs, healthcare professionals	Developing a healthcare hackathon to address unmet clinical needs	Quantitively/ questionnaire	The high impact of healthcare hackathons on participant perceptions and attitudes toward medical innovation and interdisciplinary collaboration	Hackathon	High
14	Shaheen S. Saffari [23]	December 2018	BMC medical education	United States of America	2 months	Quantitative	20	Students, faculty	Developing a hackathon to create an original dental curriculum	Quantitively/ questionnaire	Providing a view to the integration of basic science into clinical instruction, evidence-based dentistry, inter-professional education, the need to educate the communication, leadership, conflict management skills	Hackathon	High
15	Atipong Pathanasethpong [36]	October 2017	JMIR mHealth and uHealth	Thailand	1 month	Quantitative	80	Students, faculty	Developing a hackathon to solve public health issues	Quantitively/ questionnaire	Providing insight into the feasibility and benefits of interdisciplinary collaboration and innovation in Health, addressing cultural differences, and managing large numbers of mentors	Hackathon	Moderate
16	Abdulhammed Opeyemi Babatunde [37]	January 2023	medRxiv	Africa	3 days	Quantitative	50	Students	Developing a hackathon to address societal problems	Quantitively/ questionnaire	A positive experience for participants. resulting in ten public health innovations and prototypes	Hackathon	High
17	Milton Alberto Muñoz-Leija [38]	April 2021	European Journal of Anatomy	Canada	-	Quantitative	-	Students	Developing a hackathon to improve medical education	Quantitively/ questionnaire	Emphasizing the importance of a multidisciplinary team and the use of technology for solving problems in medical education	Hackathon	Low
18	Waqaas Akmal Butt [29]	August 2021	Surgical Innovation	Pakistan	1 month	Quantitative	109	Nurses, doctors, students, IT professionals, engineers, business people, public health specialists	Developing a hackathon to find efficient and novel solutions to problems in surgery	Quantitively/ questionnaire	High scores for project relevance and participants’ willingness to continue working on the projects after the event	Hackathon	High
19	Nensi M. Ruzgar [24]	September 2020	Journal of Surgical Education	United States of America	1 day	Quantitative	31	Students, surgeons	Developing a hackathon to address concerns faced by surgeons and trainees in terms of diversity and sustainability in surgery and surgical education	Quantitively/ questionnaire	Creating innovative and sustainable solutions to surgical workforce concerns	Hackathon	High
20	Kirsten Cooper [25]	March 2018	Journal of the American College of Radiology	United States of America	-	Qualitative	200	Physicians, students, industry members	Developing a hackathon to problem-solve broad issues within radiology, creating an integrative environment for collaboration	Qualitatively/ expert judgment	providing an opportunity for radiologists and other participants to gain real-time experience exploring the complex task of innovating new products and services while working within a diverse team	Hackathon	Low
21	Julie K. Silver [26]	July 2016	Journal of Medical Systems	United States of America	2 days	Qualitative	150	Physicians, healthcare professionals, data scientists, engineers, user interface designers, business professionals, students	Developing a hackathon to accelerate the innovation of medical solutions, improve the design in the beginning stages, and support educational training	Qualitatively	Discussing hackathons as a platform for interdisciplinary education and promoting innovation in the healthcare industry	Hackathon	Moderate
22	R. Cameron Craddock [27]	December 2016	GigaScience	United States of America	2 years	-	-	Students, researchers	Developing a hackathon promotes open, cross-institutional, and interdisciplinary collaboration in neuroscience research	-	Resulting in disciplinary collaborations to improve data collection, the development or optimization of data analysis tools, testing hypotheses about brain structure using openly shared data	Hackathon	Moderate

## Discussion

This scoping review provided a detailed overview of the existing literature on hacking in health profession education and explored what we know (and do not know) about this emerging trend. Our results emphasized the increasing pattern of utilizing hacking in HPE for enhancing teaching and learning, problem-solving, and product generation. Our findings revealed that elements of hacking in HPE can include; innovation, creativity, critical thinking, and collaboration. Innovation is a critical element of hacking in education that holds different meanings for different disciplines. Those involved in HPE consider innovation to create new tools and resources [7, 28], hackathons [13, 19, 20, 22–38], gamification [28], and simulation-based training [21].

This study by introducing a different perspective or a new application of hacking that has not been explored before allows for a broader understanding of hacking and its potential positive applications in HPE. Although it does mention “hacking,” it does not refer to the malicious or illegal activities often associated with the term [43, 44]. The results of this study indicate incorporating hacking into HPE aimed at improving education and enhancing clinical or healthcare had positive outcomes in learning, attitudes, knowledge, and skills. Embracing hacking in HPE revolutionizes traditional teaching methods, promotes interdisciplinary collaboration, leverages cutting-edge technologies, and cultivates a culture of lifelong learning, ultimately enhancing clinical outcomes and the healthcare system as a whole [13, 20–22, 26–28, 30–34, 36–38].

This study reveals that hackathons are more prominent in the United States of America (USA) education system compared to other countries due to the culture of innovation and entrepreneurship [7, 19–28]. It is important to note that while hackathons are more prominent in the USA, they are also gaining popularity in other countries [13, 29–38]. This mindset directly contributes to designing effective interventions and driving innovation across different countries and regions around the world. In comparison to other educational interventions, in hacking within education studies, the geographical context, the focus of the intervention, and outcomes can play a significant role in shaping the educational intervention. The relationship between them can be explained through Socio-cultural theory which emphasizes the influence of social interactions and cultural factors in learning and development [45]. According to this theory, factors such as cultural values, societal norms, availability of technological resources, access to educational opportunities, and collaboration with local communities all play a role in shaping the outcomes of hacking in education. In light of the findings, creating a positive impact on education through “hacking” as innovation requires adaptations and overcoming challenges. Adaptations could involve modifying traditional teaching methods, incorporating new technologies into the learning process, or adopting new pedagogical approaches, such as project-based learning or blended learning [40]. Adapting education through hacking means finding innovative solutions to improve teaching methods, student engagement, and overall learning outcomes [46]. Challenges refer to the obstacles or barriers that educators, leaders, or organizations may face when trying to implement innovative changes in education could be related to resistance to change, lack of resources or funding, bureaucratic hurdles, or simply the complexities of navigating a rapidly changing educational landscape [47]. Therefore, driving positive change requires leading with creativity, perseverance, and collaboration [48]. In this way, different leadership and management approaches and models can help to create change. For example, studies show that Kotter’s 8-Step Change theory can be considered a guide for educators to lead innovation in education through hacking [49].

With a clear definition of innovation, the next is to consider how to systematize and embed a culture of innovation within the educational organization. An important component of this strategy is tying innovation to professional, school, and university priorities. Innovation is a human-centered endeavor and requires key stakeholders’ engagement to identify challenges and opportunities. Our findings emphasized that while meeting with multiple stakeholders is critical, developing other champions of an innovation focus is essential. Consider resources available in developing internal and external advisory members, local entrepreneurs, or leaders in innovation roles. Other strategies can be used to guide the design and development of innovation programs including co-design sessions, focus groups, and the use of external consultants.

Faculty members are the main actors of change and the most effective source of creativity in education. They have a significant role to play in driving change in education by preparing the ground for creativity, adapting to new changes, and stimulating change within the classroom. They can create a positive and innovative learning environment that benefits both students and the entire organization [50, 51].

For many faculty members, innovation will be a new area of inquiry. Hence, based on our findings we recommend to the planners and organizers of faculty development programs to design and implement some programs about innovation in the teaching and learning process considering these three key elements: building knowledge, acquiring skills in applying rigorous innovation methodologies to identifying and solving problems, and generating opportunities to participate in innovation activities can way to develop an interest in innovation and elevate it as a school goal and priority [51, 52].

Overall, these findings demonstrate that the hackathon effectively met its objectives in the case of HPE by promoting interdisciplinary collaboration, building relationships, facilitating learning, developing innovation, knowledge acquisition, practical problem-solving skills, cross-disciplinary tools for teaching and learning, and inquiry-based learning. In addition, findings reveal the positive outcomes of hackathons in HPE including increasing confidence levels as innovators, enhancing awareness of technological possibilities for future healthcare givers, improved familiarity with healthcare innovation and teaching entrepreneurship, improving engagement, and learning outcomes in training, high participant satisfaction, and increased motivation with the program. Also, Hackathon in HPE emphasizes the role of multidisciplinary teams and technology in solving medical education problems and encourages disciplinary collaborations to improve data collection and analysis [7, 13, 19–38]. A potential gap of knowledge in this study is the lack of research on the long-term impact and sustainability of hacking in HPE. While the study highlights the positive outcomes of incorporating hacking into education, it does not delve into the long-term effects or address the potential challenges in maintaining and sustaining these innovative practices. Additionally, there is limited mention of the assessment methods used to measure the effectiveness of hacking in education, which could be an area for further investigation.

Some limitations of this study are including, this comprehensive study includes a straightforward research question, a predefined search strategy, and inclusion and exclusion criteria for studies that summarize all relevant studies, allowing for a detailed understanding of the available evidence. This had some limitations when it came to collecting eligible articles. Since this review extracted only published research, there are educational interventions that are reported at conferences but have not yet been published in the literature. The moderate quality of full-text studies is indeed a limitation of this study. Future research should consider including higher-quality full-text studies to enhance the robustness of the findings.

Although we searched for articles using general keywords, these were limited to hackathon keywords. Further research is needed to conduct hackathons in HPE to drive sustained innovation and crowd-source solutions. First, research should investigate how to enhance faculty and student engagement and retention to foster hackathons in HPE. Second, a multidisciplinary study is crucial to strike a balance between embracing innovation and evaluating its impact to ensure its successful integration into the education system. Third, future research could focus on exploring the long-term impact, sustainability, and assessment methods of incorporating hackathons in HPE.

## Conclusion

Hacking in the health profession educational context refers to the positive applications in teaching and learning that have not been explored before. Embracing hacking requires adaptations, overcoming challenges, and driving change through creativity, perseverance, and collaboration. The goal of hacking in health profession education is to create a more dynamic, adaptable, and effective educational system that meets the needs of all learners and prepares them for success in the rapidly evolving 21st-century economy.

### Electronic supplementary material

Below is the link to the electronic supplementary material.

Supplementary Material 1

## Data Availability

The datasets used and/or analyzed during the current study are available from the corresponding author on reasonable request.
