# Supplementary material for: Health profession education hackathons: a scoping review of current trends and best practices
Source: BMC Med Educ. 2024 May 21;24:554. doi: 10.1186/s12909-024-05519-7 (PMC11110329; doi:10.1186/s12909-024-05519-7)
Supplement: Supplementary file 1 — Supplementary Material 1 [file 12909_2024_5519_MOESM1_ESM.docx]

**Appendix 1. Search strategy**

**1. PubMed**

hack*[tiab] AND (“Education, Professional”[mesh] OR “medical education” [tiab] OR “medical training”[tiab] OR “nursing education”[tiab] OR “dental education”[tiab] OR “pharmacy education”[tiab] OR “health professions education”[tiab] OR “health professional education”[tiab] OR “higher education”[tiab] OR “healthcare education”[tiab] OR “health care education”[tiab] OR “Students, Health Occupations”[mesh] OR “medical student*”[tiab] OR “nursing student*”[tiab] OR “dental student*”[tiab] OR “pharmacy student*”[tiab] OR "Schools, Health Occupations"[Mesh] OR “medical school*”[tiab] OR “nursing school*”[tiab] OR “dental school*”[tiab] OR “pharmacy school*”[tiab])

**53**

**2. EMBASE**

hack*:ab,ti AND ('medical education'/exp OR 'medical training':ab,ti OR 'nursing education':ab,ti OR 'dental education':ab,ti OR 'pharmacy education':ab,ti OR 'health professions education':ab,ti OR 'health professional education':ab,ti OR 'higher education':ab,ti OR 'healthcare education':ab,ti OR 'health care education':ab,ti OR 'health student'/exp OR 'medical student?':ab,ti OR 'nursing students?':ab,ti OR 'dental student?':ab,ti OR 'pharmacy student?':ab,ti OR 'medical school'/exp OR 'pharmacy school'/exp OR 'university'/exp OR 'medical school?':ab,ti OR 'nursing school?':ab,ti OR 'dental school?':ab,ti OR 'pharmacy school?':ab,ti)

**97**

**3. Scopus**

TITLE-ABS-KEY(hack*) AND TITLE-ABS-KEY(“medical education” OR “medical training” OR “nursing education” OR “dental education” OR “pharmacy education” OR “health professions education” OR “health professional education” OR “higher education” OR “healthcare education” OR “health care education” OR “medical student?” OR “nursing student?” OR “dental student?” OR “pharmacy student?” OR  “medical school?” OR “nursing school?”  OR “dental school?” OR “pharmacy school?”)

**200**

**4. Web of Science**

Science Citation Index Expanded (SCI-EXPANDED), Social Sciences Citation Index (SSCI), Emerging Sources Citation Index (ESCI), Conference Proceedings Citation Index – Science (CPCI-S), Conference Proceedings Citation Index – Social Science & Humanities (CPCI-SSH)

TS=(hack*) AND TS=(“medical education” OR “medical training” OR “nursing education” OR “dental education” OR “pharmacy education” OR “health professions education” OR “health professional education” OR “higher education” OR “healthcare education” OR “health care education” OR “medical student?” OR “nursing student?” OR “dental student?” OR “pharmacy student?” OR “medical school?” OR “nursing school?” OR “dental school?” OR “pharmacy school?”)

**113**

**5. ERIC (ProQuest)**

ti,ab(hack*) AND ti,ab(“medical education” OR “medical training” OR “nursing education” OR “dental education” OR “pharmacy education” OR “health professions education” OR “health professional education” OR “higher education” OR “healthcare education” OR “health care education” OR “medical student?” OR “nursing student?” OR “dental student?” OR “pharmacy student?” OR “medical school?” OR “nursing school?” OR “dental school?” OR “pharmacy school?”)

**40**

**6. APA PsycINFO (ProQuest)**

ti,ab(hack*) AND ti,ab(“medical education” OR “medical training” OR “nursing education” OR “dental education” OR “pharmacy education” OR “health professions education” OR “health professional education” OR “higher education” OR “healthcare education” OR “health care education” OR “medical student?” OR “nursing student?” OR “dental student?” OR “pharmacy student?” OR “medical school?” OR “nursing school?” OR “dental school?” OR “pharmacy school?”)

**36**

**7. Education Source (EBSCOhost)**

(TI (hack*) AND TI (“medical education” OR “medical training” OR “nursing education” OR “dental education” OR “pharmacy education” OR “health professions education” OR “health professional education” OR “higher education” OR “healthcare education” OR “health care education” OR “medical student?” OR “nursing student?” OR “dental student?” OR “pharmacy student?” OR “medical school?” OR “nursing school?” OR “dental school?” OR “pharmacy school?”)) OR (AB (hack*) AND AB (“medical education” OR “medical training” OR “nursing education” OR “dental education” OR “pharmacy education” OR “health professions education” OR “health professional education” OR “higher education” OR “healthcare education” OR “health care education” OR “medical student?” OR “nursing student?” OR “dental student?” OR “pharmacy student?” OR “medical school?” OR “nursing school?” OR “dental school?” OR “pharmacy school?”))

**90**

**8. CINAHL Plus with Full Text (EBSCOhost)**

(TI (hack*) AND TI (“medical education” OR “medical training” OR “nursing education” OR “dental education” OR “pharmacy education” OR “health professions education” OR “health professional education” OR “higher education” OR “healthcare education” OR “health care education” OR “medical student?” OR “nursing student?” OR “dental student?” OR “pharmacy student?” OR “medical school?” OR “nursing school?” OR “dental school?” OR “pharmacy school?”)) OR (AB (hack*) AND AB (“medical education” OR “medical training” OR “nursing education” OR “dental education” OR “pharmacy education” OR “health professions education” OR “health professional education” OR “higher education” OR “healthcare education” OR “health care education” OR “medical student?” OR “nursing student?” OR “dental student?” OR “pharmacy student?” OR “medical school?” OR “nursing school?” OR “dental school?” OR “pharmacy school?”))

**16**

| 1 | PubMed | 53 |
| --- | --- | --- |
| 2 | Embase | 97 |
| 3 | Scopus | 200 |
| 4 | Web of Science | 113 |
| 5 | ERIC | 40 |
| 6 | PsycINFO | 36 |
| 7 | Education Source | 90 |
| 8 | CINAHL | 16 |
| Total | | **645** |
